# Case study on impact performance optimization of hydraulic breakers

**DOI:** 10.1186/s40064-016-2769-1

**Published:** 2016-07-16

**Authors:** Dae-Kyung Noh, Young-Ky Kang, Jae-Sang Cho, Joo-Sup Jang

**Affiliations:** School of Mechanical Engineering, Gachon University, 1342, Seongnamdaero, Seongnam-si, Gyeonggi 13120 South Korea; R&D Center, Soosan Heavy Industries Co., LTD., 260, Jeongmunsongsan-ro, Hwaseong-si, Gyeonggi 18628 South Korea

**Keywords:** Impact frequency, Impact energy, Pressure pulsation, Multiobjective function, Optimization

## Abstract

**Background:**

In order to expand the range of activities of an excavator, attachments, such as hydraulic breakers have been developed to be applied to buckets. However, it is very difficult to predict the dynamic behavior of hydraulic impact devices such as breakers because of high non-linearity. Thus, the purpose of this study is to optimize the impact performance of hydraulic breakers. The ultimate goal of the optimization is to increase the impact energy and impact frequency and to reduce the pressure pulsation of the supply and return lines.

**Results:**

The optimization results indicated that the four parameters used to optimize the impact performance of the breaker showed considerable improvement over the results reported in the literature. A test was also conducted and the results were compared with those obtained through optimization in order to verify the optimization results. The comparison showed an average relative error of 8.24 %, which seems to be in good agreement.

**Conclusions:**

The results of this study can be used to optimize the impact performance of hydraulic impact devices such as breakers, thus facilitating its application to excavators and increasing the range of activities of an excavator.

## Background

Most excavators used at construction sites are equipped with buckets. However, certain tasks cannot be performed using only these buckets. Thus, attachments with various functions, such as breakers and crushers, have been developed to expand the range of tasks that excavators can perform. Among them, breakers are equipment developed to demolish targets by repetitive impact. Although a variety of studies have been conducted to analyze and improve the dynamic behavior of impact devices with hydraulic systems, no study has so far provided a practically realizable design that can be applied to real systems (Noh et al. 2014a, b; Seo et al. 2015). A hydraulic impact device such as a breaker is dominated by the repulsive properties of the impact target. It is difficult to simulate their dynamic behavior because of high nonlinearity (Oh et al. 2011). Although previous researchers have developed analysis models to predict the dynamic behavior, these models have large errors associated with supply pressure when compared with actual measurements. Because detailed pressure waveforms are highly sensitive to impact target characteristics, errors are generated; consequently, the pulsation amplification characteristics of the supply pressure that include the overall behavior of the breaker should be similar to actual measurements (Shin and Kwon 2011). Let us assume that a sensitivity analysis of the variables is carried out and optimum designs are obtained by utilizing an analysis model whose pulsation amplitudes of the supply pressure are completely different from the measured values. If a prototype is manufactured according to designs that are obtained as described above, it is not likely to meet the expected performance.

Optimization case studies of current commercial breaker products show that the impact energy and impact frequency have opposite characteristics (Ryoo and Chang 2010). That is, the impact frequency decreases if the impact energy increases. The impact output, which is a core element that determines the impact performance, is a multiplication form of the impact energy and impact frequency. However, no true performance improvement is achieved if the optimization results indicate that the impact energy increases while the impact frequency decreases.

In this study, an analysis model that produces a supply pressure similar to the actual measured value is developed, and the model is used to conduct case studies that involve a sensitivity analysis of the variables and performance improvement optimization.

## Development of analysis model of breaker and impact performance optimization

In general, an optimization procedure utilizing computer-aided engineering is divided into the following steps: the development of an analysis model, reliability verification, a sensitivity analysis of the variables, the establishment of multiobjective functions, and the optimization of the multiobjective functions (performance optimization). This general optimization procedure is also adopted in this study, but the novelty of this study is the precise implementation of the supply pressure pulsation (amplitude) and the configuration of multiobjective functions that are not compromised with past case studies. The analysis tools employed are SimulationX (ITI, Germany) and EasyDesign (IDO, Korea). The mathematical modeling of breakers has already been carried out by numerous senior researchers (Choi and Chang 2009; Kwak and Chang 2008). One of these studies involved the mathematical modeling of a product whose structure was identical to that of the breaker considered in the present study. The mathematical approach employed in the previous study adopted the method used by Sung et al. (2003) by using commercial software SimulationX. For more reliable results, accurate control volumes are calculated for parts that are expected to change with pressure, and these volumes are reflected in the results. Moreover, repetitive tests are performed for the components of the hydraulic fluid transfer system such as pumps and pipes. These results are reflected directly in the analytical model, thus minimizing miscellaneous errors other than those arising from modeling the breaker.

### Modeling and verification of analysis model’s reliability

**Fig. 1:**
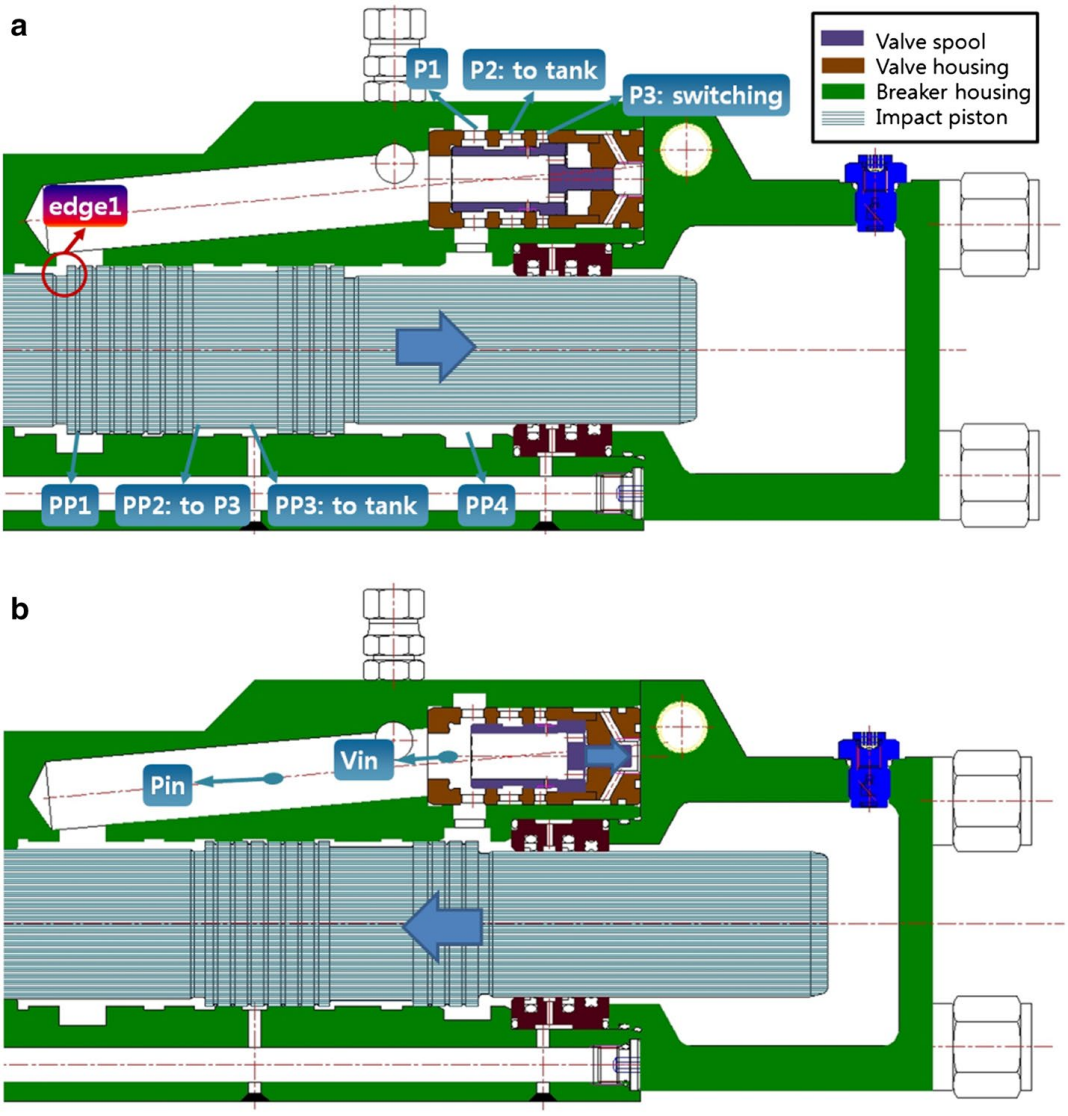
Breaker mechanism: **a** impact preparation and **b** impact

Figure 1 shows the target breaker used for the development of the analysis model. It consists of valves and an impact piston, and nitrogen gas occupies the space behind the impact piston. The impact operation can simply be divided into the following two stages.

1. Impact preparation: Fig. 1a shows the impact preparation stage. The valve spool is initially positioned such that port PP4 of the impact piston and port P2 of the valve are connected to each other. In this position, the hydraulic oil behind the impact piston can be returned to the tank. Once the hydraulic oil flows into the supply port, the valve spool maintains the initial position based on the theory of the receiving pressure area, and the impact piston is ready for impact as a force is applied in the direction of retraction.
2. Impact: Fig. 1b shows the impact stage. During the impact preparation stage, the impact piston is retracted, and piston edge 1 passes through port PP2 so that the supplied hydraulic oil flows into the switching port of the valve. The hydraulic oil introduced into the switching port is applied to the receiving pressure area, which pushes the valve spool to the right. This pushing action switches the valve, and the supplied hydraulic oil is introduced into piston port PP4 through valve port P1 so that the piston starts the impact stroke (Lee et al. 2003).

**Fig. 2:**
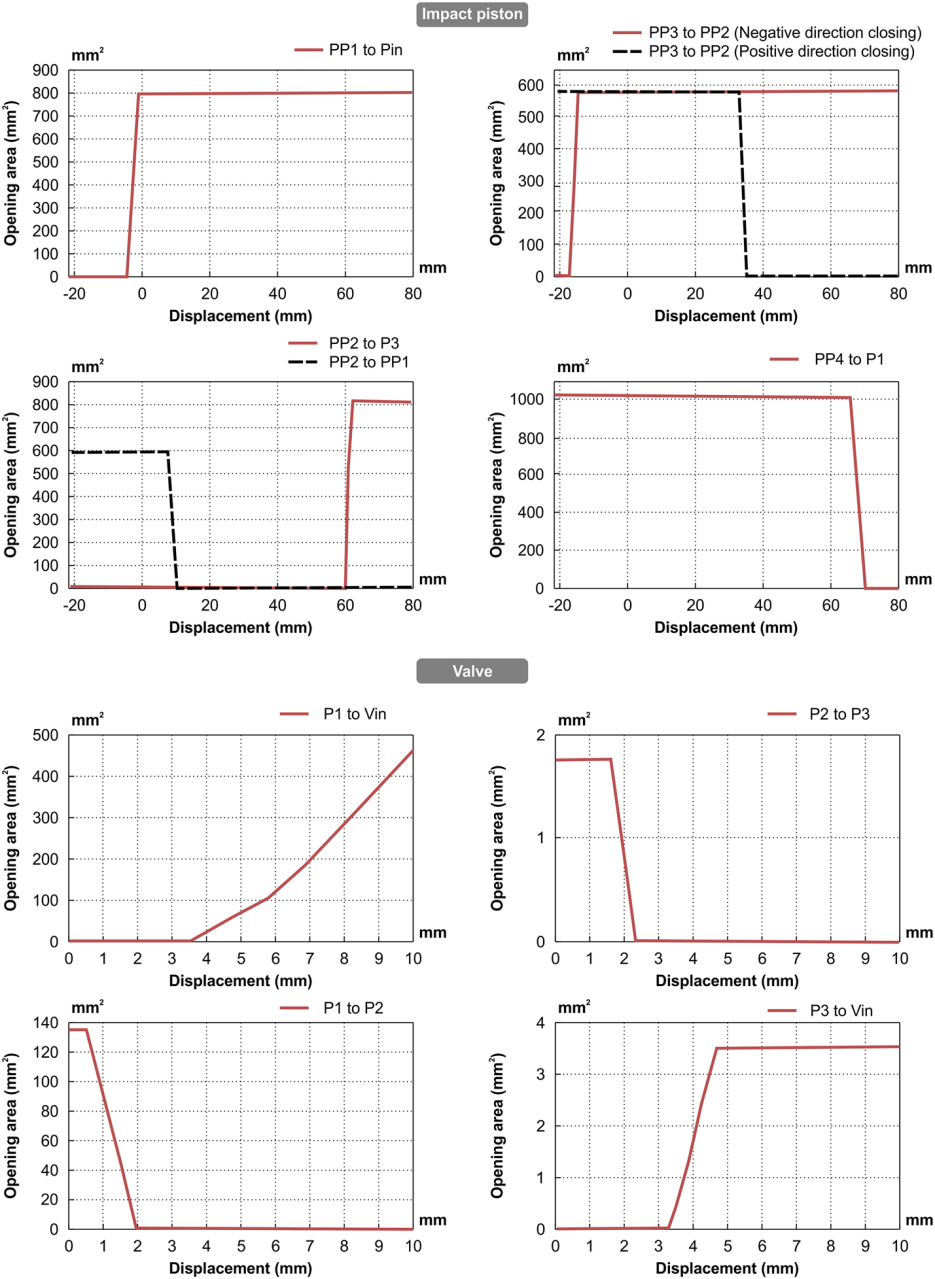
Opening area of valve spool and impact piston

**Fig. 3:**
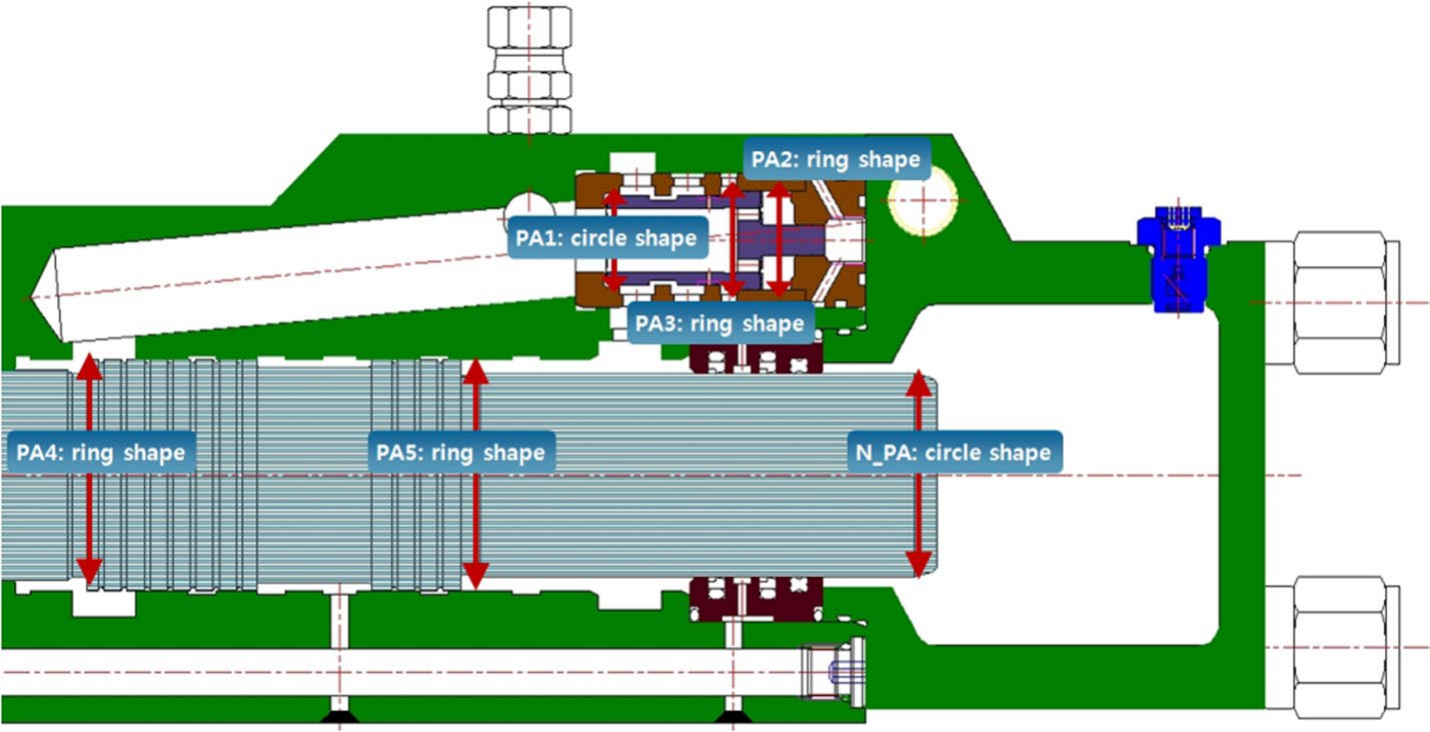
Pressure-receiving area of valve spool and impact piston

**Fig. 4:**
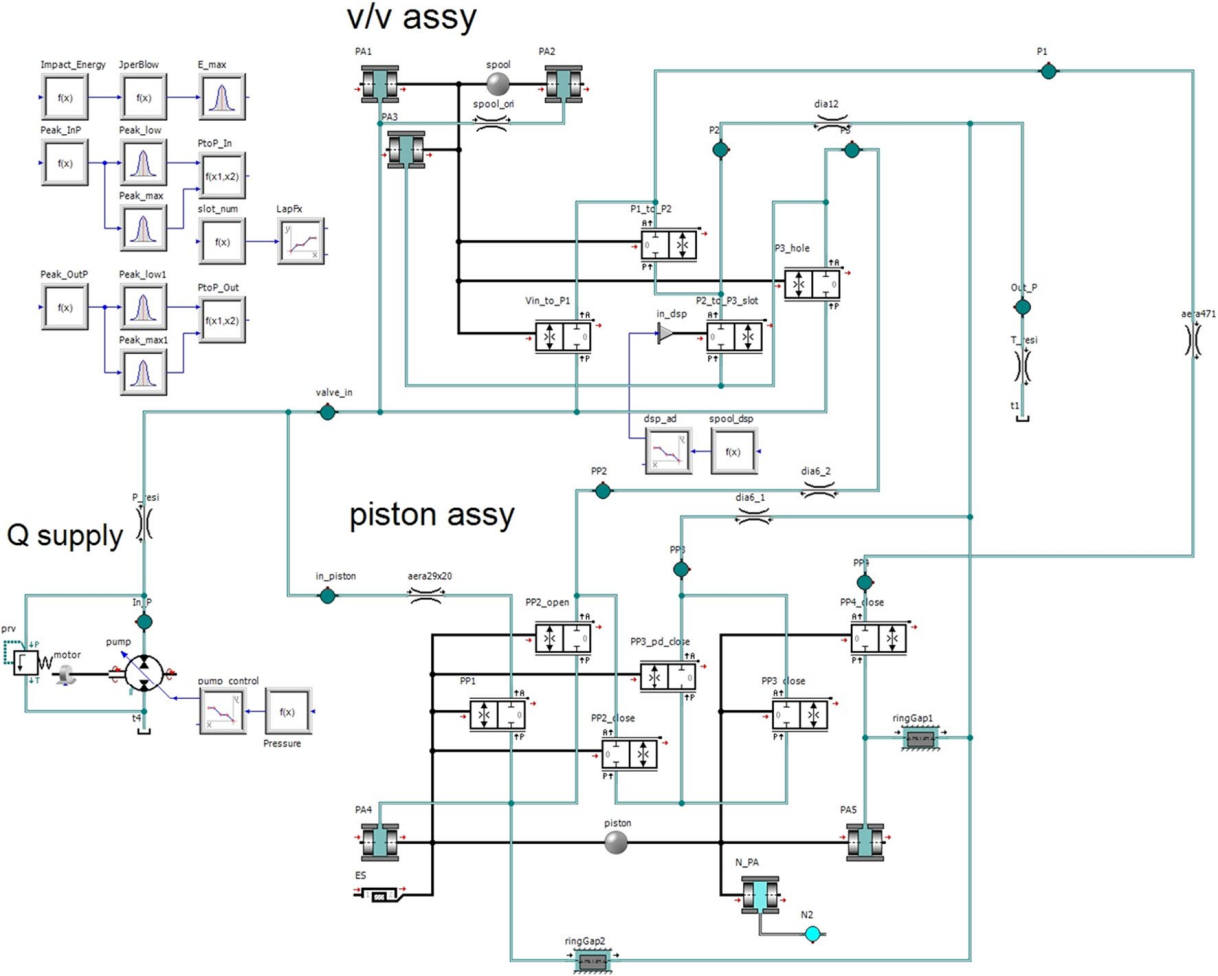
Simulation model

The dynamic analysis model of the breakers can be successfully realized if the port opening condition (in relation to the flow) and receiving pressure area (in relation to the force) are accurately implemented according to the stroke described above. Figure 2 shows the changes in the opening conditions when the positions of the impact piston and valve are changed from their initial positions in the analysis model. As shown in the figure, the opening relationship was accurately represented according to the lap condition. Figure 3 shows the receiving pressure area of the impact piston and valve. Assuming that the effect of nitrogen gas is ignored in the impact piston, the pressure applied to receiving pressure area PA5 should be 78.85 % of the pressure applied to receiving pressure area PA4 to ensure that the impact is provided only via pure hydraulic power. To enable switching of the valve, the pressure applied to receiving pressure area PA3 should be 47.09 % of the pressure applied to receiving pressure areas PA1 and PA2. The dynamic analysis model (Fig. 4) was developed using the above theories pertaining to the opening conditions and receiving pressure area.

**Fig. 5:**
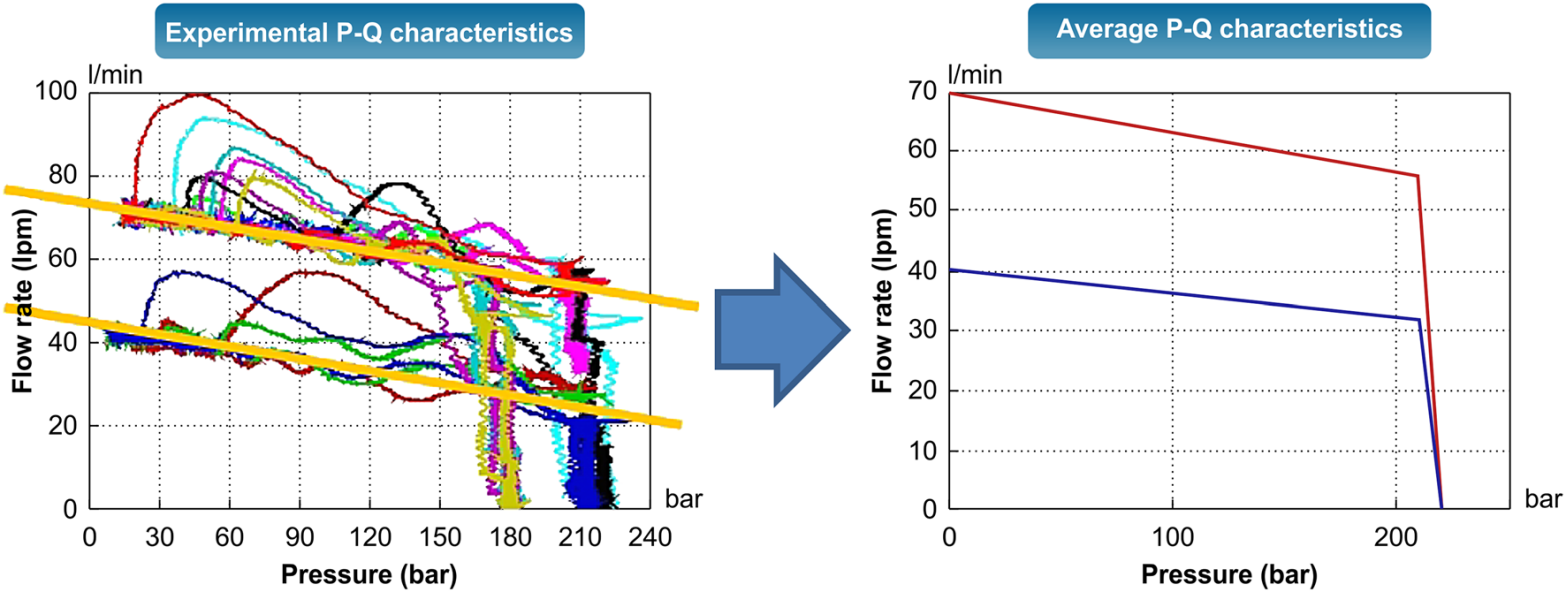
Pressure flow characteristics of excavator hydraulic oil transmission system

**Fig. 6:**
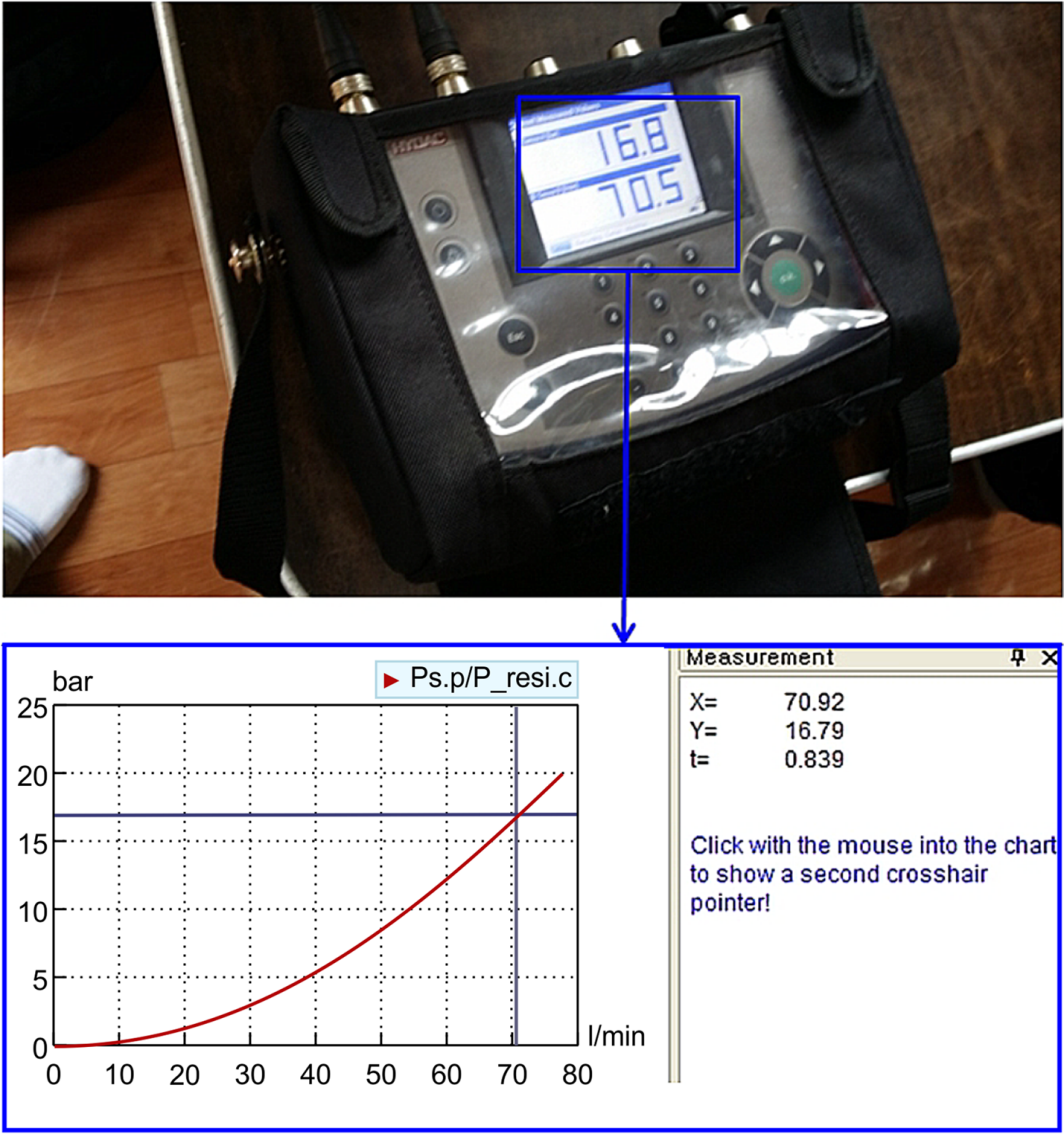
Pressure drop in inlet and outlet ports of breaker

**Fig. 7:**
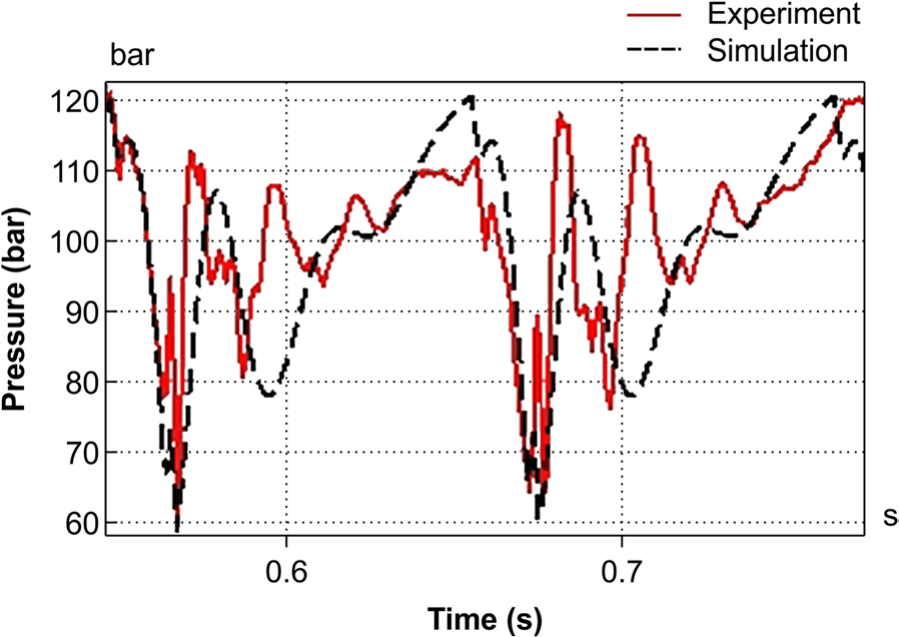
Comparison between supply pressures obtained from test and analysis

In the past, the supply pressure pulsation of breakers has been considered to be a difficult problem that cannot be described by analysis models. This is because the complicated hydraulic oil transmission system of excavators cannot be implemented in analysis models. In general, excavators rotate hydraulic pumps according to the engine’s speed, and the total volume of the discharged hydraulic oil is divided and transferred into each operating part. Such transmission systems are highly complicated, and it takes a considerable amount of time to implement such systems using analysis models. In this study, the time required for the modeling process was reduced by directly applying actual machine test results to the analysis models, and the errors that could possibly occur in each transmission system were minimized (Kwak and Chang 2008). Figure 5 shows the P–Q characteristics obtained by taking the characteristics of the transmission systems of excavators into consideration. The characteristics of the discharged flow were tested according to the loads and mean values reflected in the analysis models by adjusting the opening of the hydraulic control valves. Figure 6 shows the pressure drop characteristics of the inlet and outlet ports of the breakers. Once the characteristics of the inlet and outlet ports as well as those of the transmission system are reflected, the resultant model can be considered to be equivalent to an excavator system without the breaker. Figure 7 shows a comparison between the supply pressure pulsation obtained using the analysis model and that obtained from a breaker test performed at a supply flow of 40 lpm and a relief valve set pressure of 210 bar. Because the supply pressure pulsation amplitudes obtained from the analysis and test were approximately equal, and the impact cycle was also approximately the same as those obtained in previous case studies, it can be said that the proposed analysis model is reliable.

### Sensitivity analysis of design variables

**Fig. 8:**
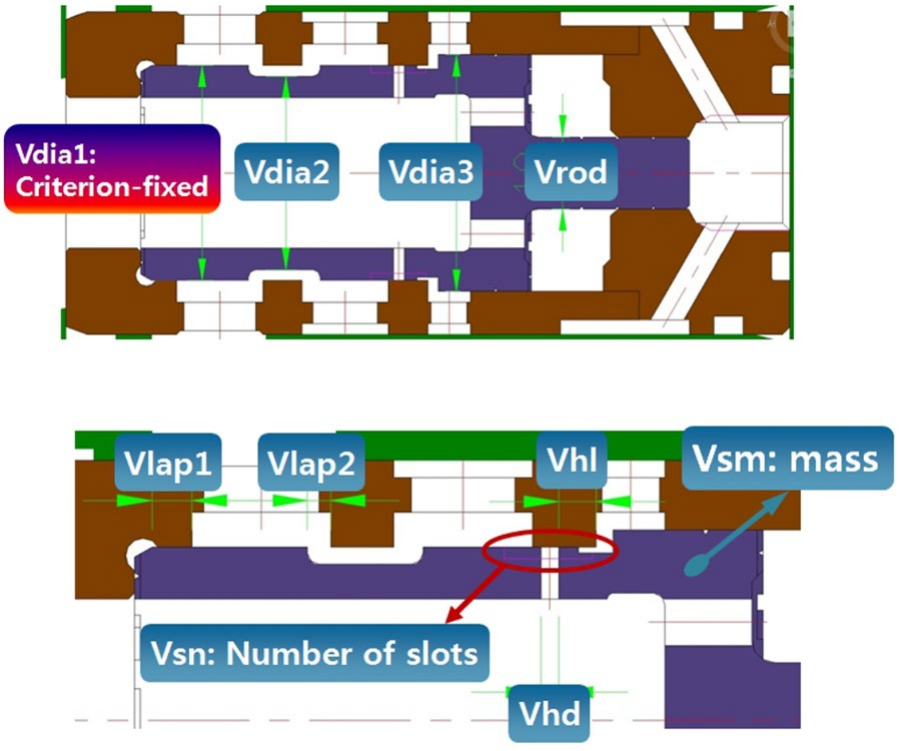
Target of sensitivity analysis of variables (valve)

**Fig. 9:**
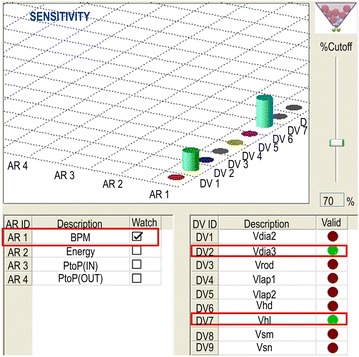
Example of high-sensitivity selection

A breaker consists of a number of design variables, and each variable has different effects on the performance and reliability of the breaker. For performance optimization, variables with better sensitivities should be selected first. Figure 8 shows the targets of the sensitivity analysis of the valve variables. There were nine variables in total, and a sensitivity analysis was performed to evaluate the impact energy, impact frequency, and pressure pulsation amplitude of the supply and return lines. The sensitivity analysis was carried out at 3Lev. The use of 2Lev in the sensitivity analysis can reduce the time required for the sensitivity analysis but accurate results cannot be guaranteed. For example, let us assume that the impact energy increases by 10 J/blow when the outer diameter of the valve is expanded from the current level by 1 mm. If so, the impact energy is reduced by 10 J/blow when the outer diameter of the valve is decreased by 1 mm in the 2Lev sensitivity analysis. However, the above approach is not suitable because highly nonlinear breakers have a clearly different effect on the increase and decrease in the design values (Oh et al. 2013). That is, the effects on the increase and decrease in the design values should be determined by performing a sensitivity analysis at 3Lev or higher. Figure 9 shows the selection of two variables that have the highest sensitivity among the nine variables with regard to the impact frequency. The same procedure was performed to extract the variables with regard to the impact energy and pressure pulsation amplitude of the supply and return lines. Finally, the following variables of the valve were selected: Vdia2, Vdia3, Vlap1, Vlap2, and Vhl. The selected variables can be described as follows. Vdia2 refers to the diameter that is concaved area between the valves, that is, the opening from P1 to P2. If Vdia2 becomes smaller than the existing size, the opening from P1 to P2 increases, thus resulting in a smooth flow of the hydraulic fluid. However, minimizing Vdia2 does not necessarily enlarge the opening because the opening area from P1 to P2 is also related to Vlap2, in addition to Vdia2. Vlap2 refers to a lap condition, wherein the enlargement of Vlap2 leads to the expansion of the opening from P1 to P2. Vdia2 and Vlap2 both determine the opening area between P1 and P2. When this opening expands, the resistance applied to the impact piston during its retreat decreases. In simple terms, it is believed that the opening from P1 to P2 can undergo unlimited expansion if the resistance is reduced. However, such unlimited expansion will cause the impact piston to bounce up excessively, triggering the pressure pulse of the return line. Therefore, this variable must be selected while considering an appropriate size. Vdia3 is a variable pertaining to valve switching. As Vdia3 increases, the valve can be switched at lower pressures. An increase in Vdia3 increases the pressure-receiving area of the valve spool, which increases the speed of strike conversion. In other words, the force required for converting the valve decreases. Because the change in the point of conversion of the impact piston can increase the amplitude of the pressure pulse, this variable also needs to be varied in a stepwise manner instead of an extreme increase or decrease. Vlap1 is the distance required for connecting Vin to P1. If Vlap1 decreases, the hydraulic fluid flows into PP4 of the impact piston more quickly, triggering a quicker conversion of the impact piston to the impact position. This swift conversion positively influences the impact frequency. In addition, because a decrease in Vlap1can create a high pressure in PA5 of the impact piston more quickly, the impact energy also increases. However, as in the case of Vdia3, Vlap1 affects the pressure pulse negatively. Therefore, this variable, similar to other variables, needs to be adjusted to an appropriate size. Vhl refers to the distance required to inject the hydraulic fluid of Vin into the valve switching port. When the valve starts switching, the hydraulic fluid starts flowing into PP2, which is the port of the impact piston. The hydraulic fluid required to switch the valve is supplied to Vdia3 from PP2. After this, Vhl is connected to Vin. This causes the hydraulic fluid of Vin to perform valve switching, thus accelerating the conversion. Because Vhl is physically linked to Vdia3, which dominates the pressure-receiving area for valve switching, it does not affect the overall behavior and only Vhl changes.

**Fig. 10:**
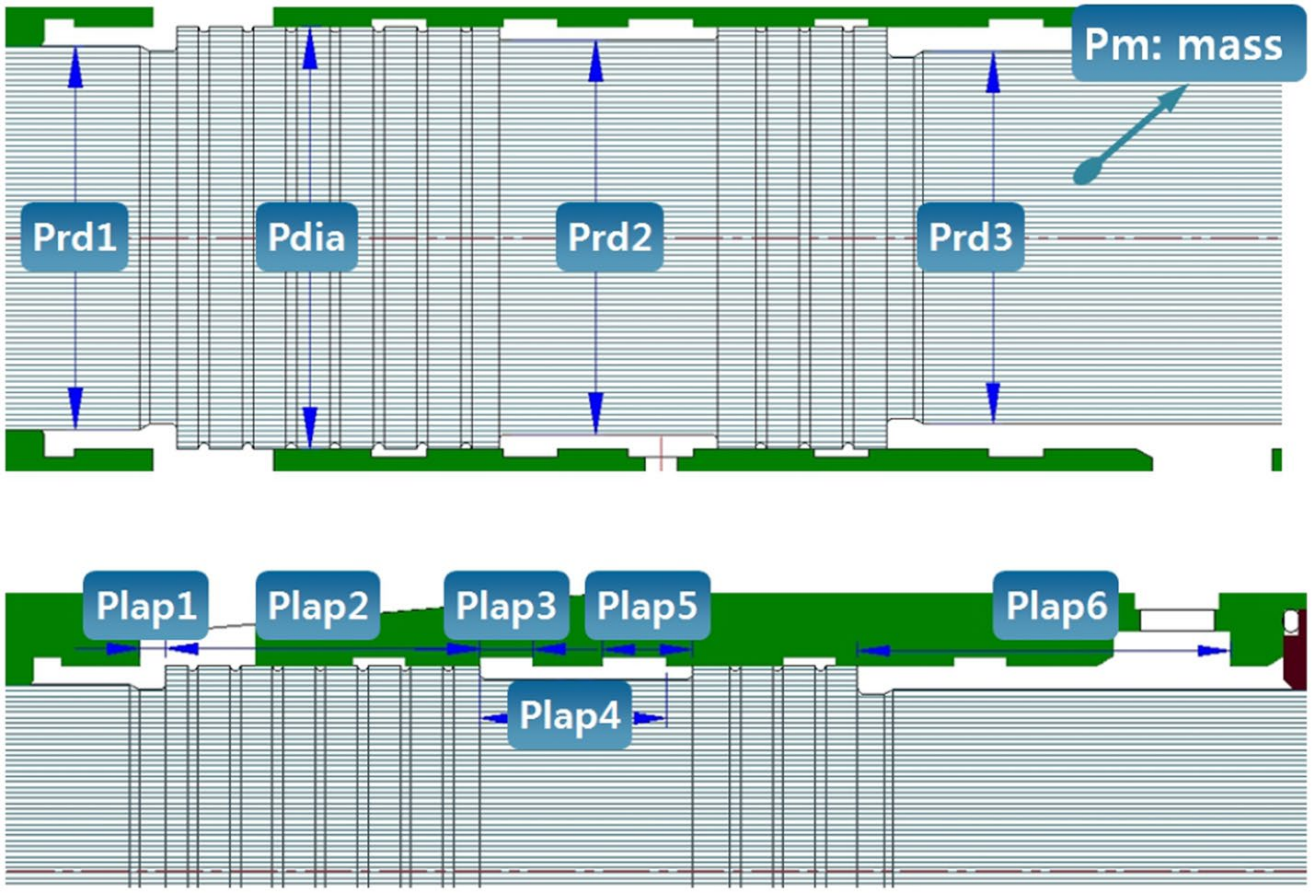
Target of sensitivity analysis of variables (impact piston)

Figure 10 shows the target variables of the sensitivity analysis of the impact piston. As in the case of the valve, each of the highest sensitivity variables were extracted with regard to the impact frequency, impact energy, and pressure pulsation amplitude of the supply and return lines for the impact piston. The selected variables were Pdia, Prd1, Prd3, Plap2, and Plap3. The selected variables can be described as follows. Pdia refers to the external diameter of the impact piston. This variable simultaneously affects PA4, which is the pressure-receiving area used for impact preparation and PA5, which is the pressure-receiving area that affects the impact movement. Reducing Pdia reduces the pressurized area, which affects the movement of the impact piston, thus increasing the force required to move the impact piston. In other words, Pdia affects the pressure-withstanding level of the supply line. In addition, because of the reduced pressurized area, the amount of fluid necessary for movement decreases. If the peak supply pressure is below the predetermined pressure of the relief valve, both the impact frequency and impact energy can be increased. However, an increase in the impact output as a single variable only can aggravate the pressure pulse. Prd1 and Prd3 are also variables related to the pressure-receiving area of the impact piston. The expansion of Prd1 causes the pressure-receiving area to decrease, thereby increasing the impact output following the same principle as Pdia. However, Prd1 affects the increase in amplitude of the pressure pulse in a different manner. The amplitude of the supply pressure pulse can be increased, whereas the amplitude of the pressure pulse of the return line decreases. The peak of the return line pulse is triggered by a momentary increase in the efflux supplied to the valve port P2 because the reaction force is applied to the impact piston directly after impact, in addition to the force applied to the pressure-receiving area PA4. However, because of the increase in Prd1, the force applied to PA4 decreases, positively affecting the return line pulse instead. Prd3 refers to a variable related to N_PA, which is the pressure-receiving area on which the pressurizing force of nitrogen gas acts. Prd3 is also related to PA5, which is the pressure-receiving area formed in the impact direction. Prd3 is applied to these two pressure-receiving areas in opposite directions. When Prd3 increases, PA5 decreases whereas N_PA increases. In other words, this variable cannot be clearly described if the force applied by the compressed nitrogen gas and the level of pressure are not clearly defined. Plap2 is related to the supply of hydraulic fluid to the valve switching port. It determines the point of conversion of the impact piston. When Plap2 decreases, the piston stroke decreases, increasing the impact frequency and reducing the impact energy because of the decrease in the acceleration area. Plap3 is related to the amount of hydraulic fluid that is supplied to the valve switching port and drawn out to the tank. As Plap3 decreases, the time required for the valve to return to its original position decreases, thus increasing the impact frequency. Moreover, because Plap3 rapidly increases the pressure of the valve switching port, the pressure pulse can also be stabilized.

Multiple variables were selected to avoid the failure of the multiobjective function that would be caused by the contradiction between variables during the optimization process.

### Impact performance optimization

The purpose of using the multiobjective function as the optimization objective is to increase the impact energy and frequency while decreasing the pressure pulsation amplitude of the supply and return lines. The optimization conditions were a supply flow of 40 lpm and a relief valve set pressure of 210 bar. Under these conditions, the objectives were to improve the supply and return line pulsation (including the impact frequency) by 15 % and the impact energy by 5 % during optimization. A lower objective value was used for the impact energy owing to the durability of the impact piston. If the impact energy is increased to a much higher value than those used in existing cases, the impact piston is likely to fracture; thus, its material should also be reviewed. Because the focus of this study was prototype manufacturing and testing, the improvement objective of the impact energy was set to a relatively low value.

Table 1 summarizes the breaker’s performance under the current design criteria, and Table 2 (impact piston) and Table 3 (valve) summarize the breaker’s performance at the minimum and maximum variances of the variables with the highest sensitivity whose selection has been described above.

**Table 1:** Performance of breaker before optimization

Impact frequency (bpm)	Impact energy (J/blow)	Pulsation (Supply) (bar)	Pulsation (Return) (bar)
540	514.60	62.54	28.44

**Table 2:** Breaker performance upon variation of high-sensitivity variables (impact piston)

Parameter	Result	Min	Max
Pdia	Impact frequency (bpm)	600	510
	Impact energy (J/blow)	544.73	488.58
	Pulsation (Supply) (bar)	70.72	66.33
	Pulsation (Return) (bar)	31.85	24.36
Prd1	Impact frequency (bpm)	540	600
	Impact energy (J/blow)	464.25	552.51
	Pulsation (Supply) (bar)	38.54	103.69
	Pulsation (Return) (bar)	32.29	23.94
Prd3	Impact frequency (bpm)	510	570
	Impact energy (J/blow)	498.71	496.56
	Pulsation (Supply) (bar)	91.34	44.22
	Pulsation (Return) (bar)	16.94	34.78
Plap2	Impact frequency (bpm)	570	540
	Impact energy (J/blow)	504.13	525.34
	Pulsation (Supply) (bar)	61.07	64.00
	Pulsation (Return) (bar)	28.26	28.59
Plap3	Impact frequency (bpm)	570	540
	Impact energy (J/blow)	514.85	514.30
	Pulsation (Supply) (bar)	60.95	64.12
	Pulsation (Return) (bar)	24.43	28.47

**Table 3:** Breaker performance upon variation of high-sensitivity variables (valve)

Parameter	Result	Min	Max
Vdia2	Impact frequency (bpm)	540	540
	Impact energy (J/blow)	514.46	514.98
	Pulsation (Supply) (bar)	62.50	62.67
	Pulsation (Return) (bar)	28.42	28.47
Vdia3	Impact frequency (bpm)	510	540
	Impact energy (J/blow)	514.42	514.71
	Pulsation (Supply) (bar)	67.83	70.85
	Pulsation (Return) (bar)	17.39	30.52
Vlap1	Impact frequency (bpm)	570	540
	Impact energy (J/blow)	517.08	510.14
	Pulsation (Supply) (bar)	75.35	68.42
	Pulsation (Return) (bar)	29.13	21.24
Vlap2	Impact frequency (bpm)	540	540
	Impact energy (J/blow)	514.00	515.32
	Pulsation (Supply) (bar)	80.00	72.48
	Pulsation (Return) (bar)	22.98	31.49
Vhl	Impact frequency (bpm)	540	540
	Impact energy (J/blow)	514.60	514.60
	Pulsation (Supply) (bar)	62.86	62.28
	Pulsation (Return) (bar)	28.13	28.69

No variables satisfied the multiobjective function objectives, except for the minimization of Plap3 (Table 2). This result implies that a contradiction between the aforementioned variables is inevitable if the optimization is performed with these variables. Thus, it is preferable to perform the optimization by selecting multiple variables simultaneously.

Ten variables with the highest sensitivity were selected as the optimization target variables. In previous case studies (Shin and Kwon 2011; Ryoo and Chang 2010), optimization results showed that the impact energy increased while the impact frequency decreased. However, in this study, a multiobjective function was designed such that both the impact energy and frequency increased.

The impact energy and impact frequency were factors related to the output, and most machines destabilize if the outputs increase (Choi and Chang 2009). Even if a very strong output is obtained but stability is not ensured, a contradiction can occur between durability and performance. Thus, to obtain a stabilized output, the multiobjective function included a reduction in the pressure pulsation amplitude of the supply and return lines.

**Fig. 11:**
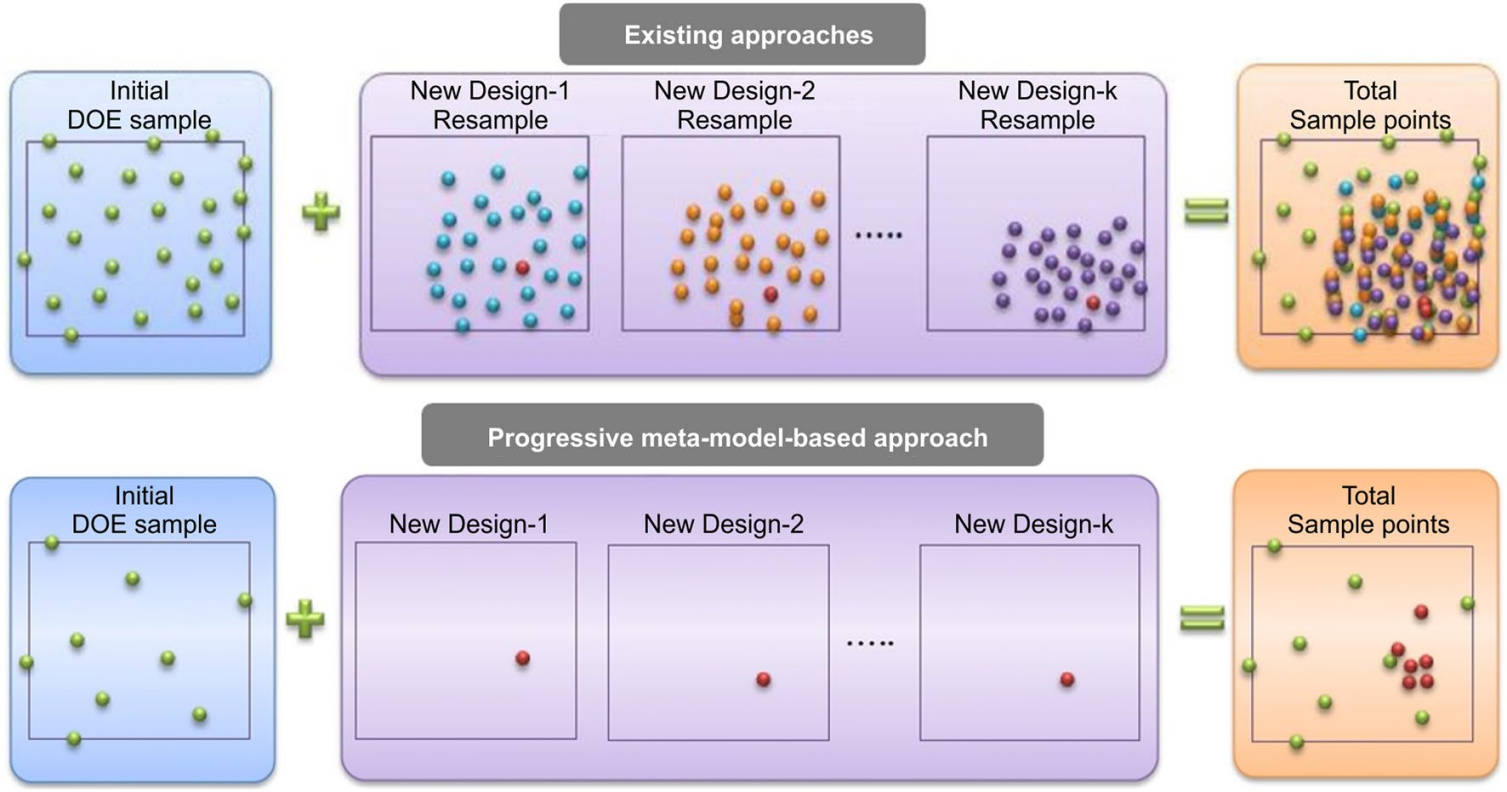
Progressive meta-model-based approach

This study followed the progressive meta-model-based optimization process, which was divided into initial and iteration processes. In the initial optimization process, incomplete small composite design-II was used to create a sample point, and during the iteration process, the augmented Lagrange multiplier algorithm was applied. Because the progressive meta-model-based optimization can only add new approximate optimum solutions to the initial sample point during the iteration process, the optimization objective can be achieved with the least number of iterations. Figure 11 shows a schematic of the progressive meta-model-based approach. The figure shows that in this approach, the number of times data collection needs to be carried out to approach the optimization objective was reduced significantly when compared with existing approaches (EasyDesign, Institute of Design Optimization, Inc.).

**Fig. 12:**
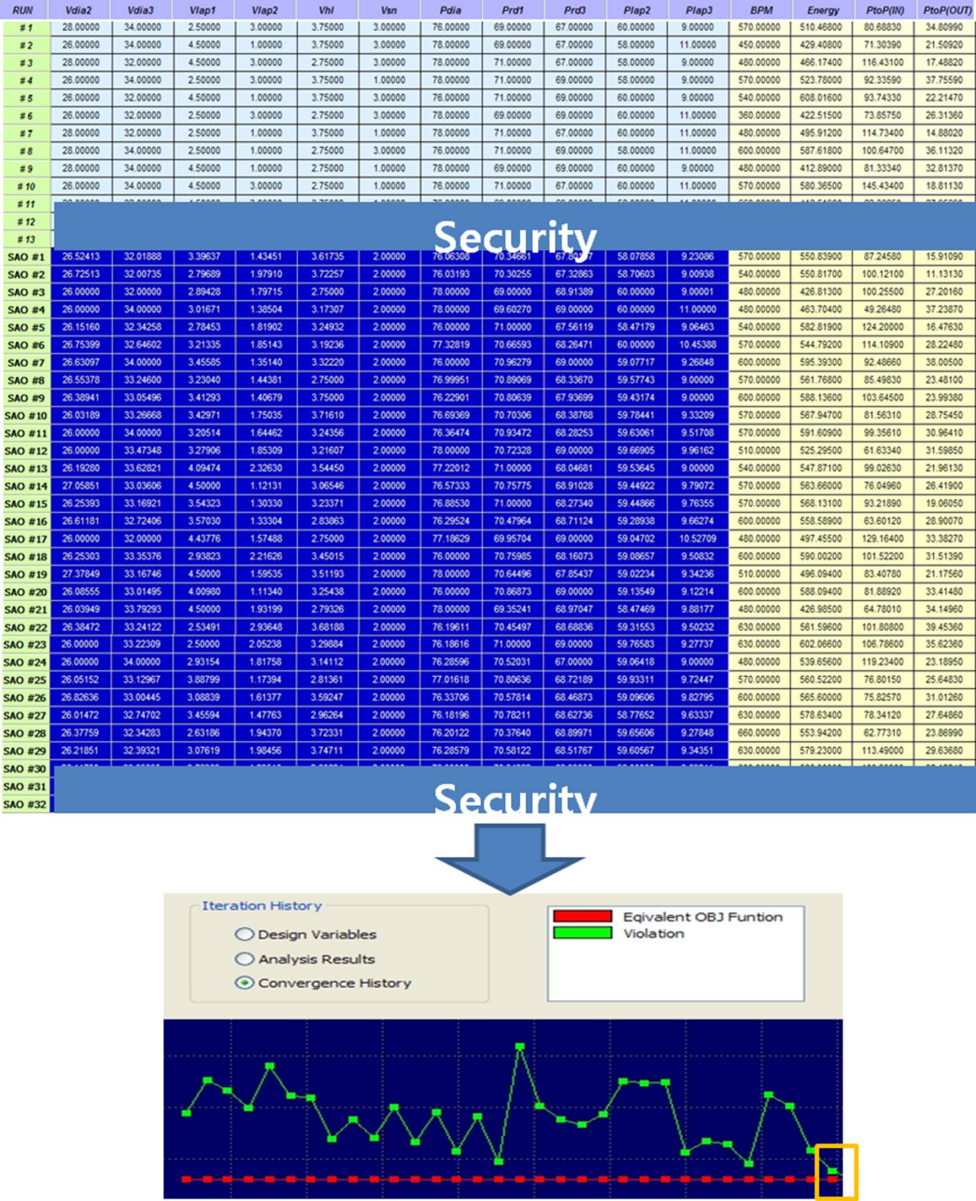
Optimization process and convergence history

Figure 12 shows the optimization process and convergence history. As shown in the figure, the objective function can be satisfied with 32 optimization iterations. In this study, the minimum variance limit of the design variable was reduced up to 0.01 μm to consider the effect of minimum changes in the design variables during the optimization process. However, if a prototype is manufactured in the future, the processability of the dimension of 0.01 μm, which was used in the current optimization, should be reestablished in consideration of the smallest dimension that can be currently processed, which is 0.1 mm.

**Fig. 13:**
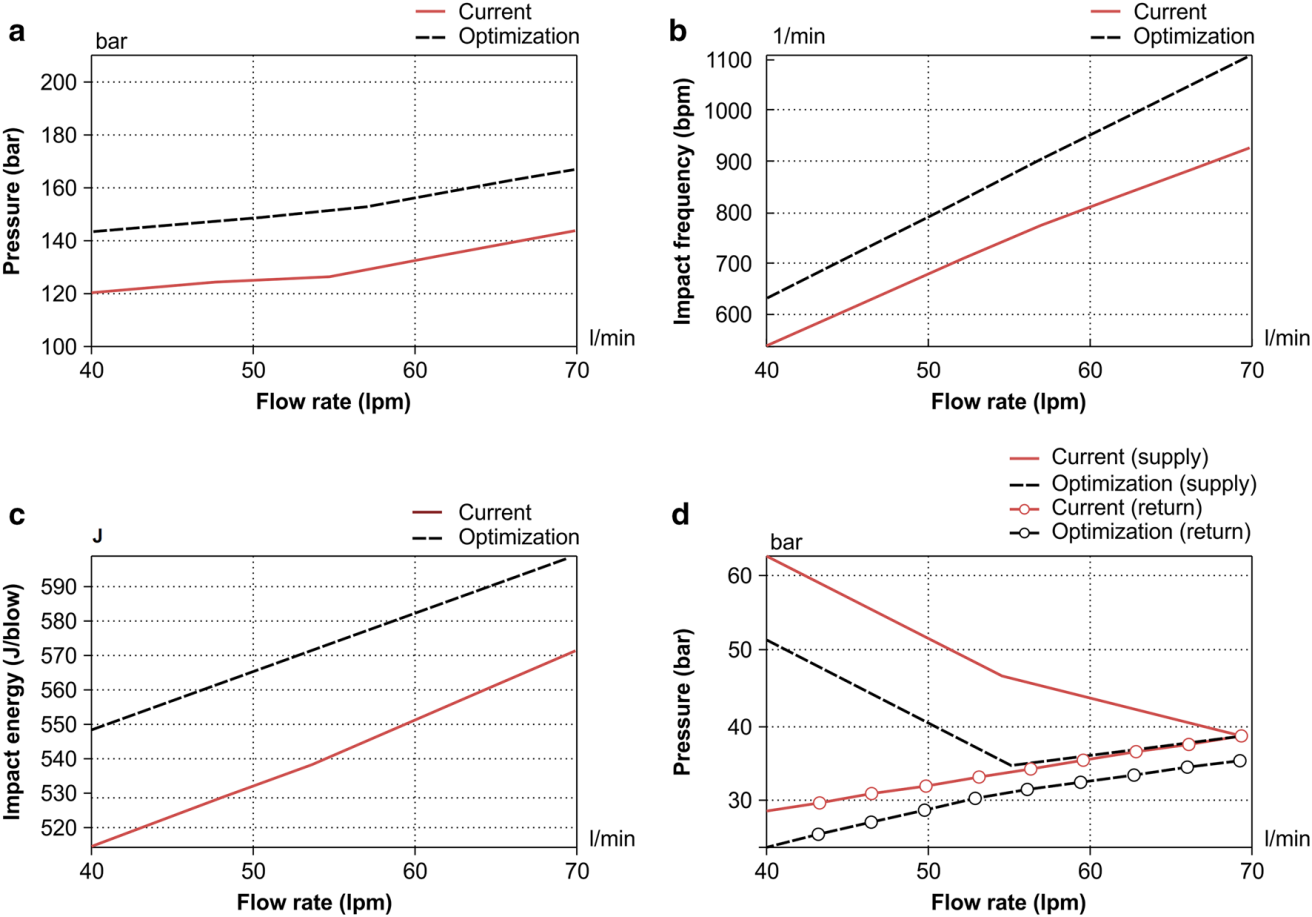
Comparison of performance before and after optimization. **a** Comparison of pressure peak before and after optimization, **b** comparison of impact frequency before and after optimization, **c** comparison of impact energy before and after optimization, and **d** comparison of pressure pulsation before and after optimization

Figure 13 shows a comparison of the performance before and after optimization when the dimensions ensuring processability are applied. The optimization process was carried out by limiting the supply flow condition to 40 lpm, but the recommended usage for the flow of the breakers in this study was up to 70 lpm. Thus, three flow conditions—40, 55, and 70 lpm—were employed to verify the optimization results. The cutoff pressure of the target system was 210 bar. Before optimization, the supply pressure peak for the breaker at the maximum flow of 70 lpm was 143.64 bar. This pressure is considerably below the system cutoff pressure, and even if the pressure after optimization is higher than this pressure, it is acceptable if it is within the cutoff pressure. As shown in Fig. 13a, the peak supply pressure, even at the maximum flow after optimization, was within the system cutoff pressure, which indicates that no reduction in the impact output occurred because of the cutoff. Figure 13b, c shows changes in the impact frequency and impact energy before and after optimization. The figure shows that the impact frequency and impact energy increased under all supply flow conditions. This means that the impact output increased under all supply flow conditions. Figure 13d shows a comparison of the peak-to-peak pressure of the supply and return lines before and after optimization. Because pressure pulsation can cause system instability, it must be considered during performance optimization. The peak-to-peak supply pressure after optimization improved at 70 lpm, which was the maximum supply flow condition, whereas no such improvement was observed for the return pressure. However, because the deterioration in the pulsation of the return pressure (0.29 % or 0.11 bar) was minimal, it was considered that the stability could be maintained. Except for the peak-to-peak return pressure at the maximum flow condition, the above results reveal that the purpose of the multiobjective function was achieved well, as mentioned previously. Tables 4, 5 and 6 summarize the quantified results.

**Table 4:** Performance of breaker after optimization (40 lpm)

	Impact frequency (bpm)	Impact energy (J/blow)	Pulsation (Supply) (bar)	Pulsation (Return) (bar)
Current	540	514.60	62.54	28.44
OPT	630	548.52	51.34	23.82
Comparison (%)	16.67	6.59	−17.91	−16.24

**Table 5:** Performance of breaker after optimization (55 lpm)

	Impact frequency (bpm)	Impact energy (J/blow)	Pulsation (Supply) (bar)	Pulsation (Return) (bar)
Current	750	540.59	46.26	33.66
OPT	870	573.25	34.62	31.21
Comparison (%)	16	6.04	−25.16	−7.28

**Table 6:** Performance of breaker after optimization (70 lpm)

	Impact frequency (bpm)	Impact energy (J/blow)	Pulsation (Supply) (bar)	Pulsation (Return) (bar)
Current	930	570.97	38.52	38.68
OPT	1110	598.38	38.63	35.46
Comparison (%)	19.35	4.80	0.29	−8.32

Previous case studies have reported on an increase in impact energy while impact frequency decreased and pressure pulsation increased. However, the present study achieved the optimization objectives for the impact frequency, pressure pulsation (15 % improvement) of the supply and return lines, and impact energy (5 % improvement). Although there was a slight deterioration in the return pressure pulsation at 70 lpm, which was the maximum flow condition, it was negligible, and a more stable performance optimization than that reported in existing studies was conclusively achieved.

To verify the optimization result, a prototype reflecting the optimized sizes was manufactured. A test was performed at a supply flow of 40 lpm, which is the same condition under which the optimization was performed. Table 7 shows the comparison between the optimization results obtained through the simulation and actual test. The pressure pulse of the supply/return lines was calculated by averaging the data obtained for 20 repetitive impacts, whereas the impact frequency was obtained by performing fast Fourier transform on the repetitive impact data for 2 s. The pressure pulse obtained from the test was applied to the analytical model to calculate the speed of the impact piston, thereby deriving the impact energy. Consequently, in terms of the four objective functions, the average relative error between the test and optimization results was 8.24 %, which proved to be a good agreement. Although the expected performance was not completely realized, a clear improvement was observed. The result also satisfied the directionality of the multi objective function, indicating that the performance optimization is possibly more advanced than those proposed in other existing studies.

**Table 7:** Comparison between optimization results obtained through simulation and actual test

	Impact frequency (bpm)	Impact energy (J/blow)	Pulsation (Supply) (bar)	Pulsation (Return) (bar)
Simulation	630	548.52	51.34	23.82
Test	600	529.70	45.31	26.92
Comparison (%)	−4.76	−3.43	−11.75	13.01

## Conclusions

In this study, the impact performance of a hydraulic breaker, which is an excavator attachment, was improved by performing optimization. The study findings are summarized as follows:

1. The operation principle of breakers was analyzed, and an analysis model in which the manufacturing drawing specifications were reflected was developed.
2. The system characteristics of excavators to which the breakers are attached were identified through testing, and these characteristics were reflected in the analysis model.
3. The reliability of the analysis model was determined by comparing the analysis results and test results for the supply pressure pulsation.
4. Through a sensitivity analysis, the variables required for the performance optimization were selected.
5. Performance optimization was carried out after adding the parameters “stabilization of the pressure pulsation” and “simultaneous increases in the impact frequency and impact energy” to the multiobjective function; these parameters were not considered in previous case studies.
6. The simulation results demonstrated that the impact performance optimization results under various supply flow conditions were valid.
7. By manufacturing a test prototype that reflected the optimization result, it was verified that the four objective functions (increased impact energy, increased impact frequency, deceased supply pressure pulse, and decreased return pressure pulse) could be satisfied.
